# Imaging of ewing sarcoma: an updated analysis including presenting features, prognostic imaging biomarkers, and treatment response assessment

**DOI:** 10.1007/s00256-026-05238-8

**Published:** 2026-04-30

**Authors:** George R. Matcuk, Tanya Tivorsak, Christopher T. Watterson, Costantino Errani, Hisaki Aiba, Amandine Crombé, Paolo Spinnato

**Affiliations:** 1 Department of Imaging, S. Mark Taper Foundation Imaging Center, Cedars-Sinai Medical Center, 8700 Beverly Blvd, Ste M-335, Los Angeles, CA 90048 USA; 2 Department of Orthopedic Oncology, IRCCS Istituto Ortopedico Rizzoli, 40136 Bologna, Italy; 3 Department of Orthopedic Surgery, Nagoya City University, Nagoya, Aichi 467-8601 Japan; 4 Department of Diagnostic Oncologic Imaging, Gustave Roussy Institute, 94805 Villejuif, France; 5 Department of Musculoskeletal Imaging, Pellegrin University Hospital, 33076 Bordeaux, France; 6 SARCOTARGET Team, Bordeaux Research Institute in Oncology (BRIC) INSERM U1312 and University of Bordeaux, 33076 Bordeaux, France; 7 Diagnostic and Interventional Radiology, IRCCS Istituto Ortopedico Rizzoli, 40136 Bologna, Italy

**Keywords:** Ewing sarcoma, Bone sarcomas, Bone tumors, Radiography, Computed tomography, Magnetic resonance imaging, Nuclear medicine

## Abstract

Ewing sarcoma is a highly aggressive small round cell sarcoma primarily affecting children and adolescents. Imaging plays a central role from diagnosis to staging, treatment response assessment, and follow-up. This review synthesizes current evidence across the various imaging modalities involved at each stage of patient management, including conventional radiography, CT, MRI, and nuclear imaging, emphasizing their complementary roles. Radiographs and CT delineate bone destruction patterns, cortical breaches, and periosteal reactions, while MRI provides superior visualization of intramedullary and soft tissue extension, as well as skip lesions. Whole-body MRI and ^18^F-FDG PET/CT enable sensitive detection of metastatic disease, with PET providing metabolic biomarkers correlated with prognosis and chemotherapy response. Imaging features, including tumor volume, diffusion metrics, and changes in contrast enhancement, increasingly allow non-invasive prediction of histologic response, a key determinant of survival. Lastly, recent quantitative methods, such as radiomics and artificial intelligence, show promise for differentiating Ewing sarcoma from other sarcomas, predicting metastases, and anticipating local recurrence. By reviewing modality-specific findings, staging strategies, and response assessment tools, this article provides a practical, updated, structured framework for radiologists and oncologists to optimize diagnosis, risk stratification, and treatment planning in Ewing sarcoma, ultimately improving patient care.

## Introduction

### Epidemiology

Ewing sarcoma is a rare but highly aggressive malignant neoplasm belonging to the family of small round cell sarcomas caused by a fusion of the FET (**F**—FUS (Fused in Sarcoma); **E**—EWSR1 (Ewing Sarcoma RNA Binding Protein 1); **T**—TAF15 (TATA‑box Binding Protein Associated Factor 15) family of genes with various members of the ETS (Erythroblast Transformation Specific) family of genes [1]. The most common fusion gene is EWSR1::FLI1 (Friend leukemia integration site 1) fusion (85%), followed by EWSR1::ERG (ETS‑related gene) fusion (10%) [2, 3].

Ewing sarcoma is the second most common malignant bone tumor in children, with an incidence of approximately 1 case per 1.5 million [4]. It represents approximately 3% of all pediatric primary malignant cancers, and in the United States around 400 new cases are diagnosed annually [4–6]. The disease predominantly affects children and adolescents, with nearly 80% of patients diagnosed before the age of 20 years, and a peak incidence during the second decade of life [7].

### Clinical Features

Most clinical features depend on the anatomical location of the primary lesion. Ewing sarcoma commonly originates in the diaphysis or diaphyseal-metaphyseal region of the lower extremities (45%), followed by the pelvis (20%) and the upper extremities (10%) [8, 9]. Ewing sarcoma may have other anatomical distributions, with 10–20% of cases originating from extraskeletal lesions [4]. According to the data from the SEER (Surveillance, Epidemiology, and End Results) database (1999–2010) involving 1,163 patients, the cancer-specific survival rates for non-metastatic Ewing sarcoma at diagnosis were 86.5% at 2 years, 72.3% at 5 years, and 66.8% at 10 years [10]. Risk factors associated with poor prognosis include metastasis at presentation, tumor size larger than 10 cm, age over 20 years, poor histological response after neoadjuvant chemotherapy, and axial tumor location [10, 11].

Local inflammatory signs, fever, and constitutional symptoms can mimic infection or inflammatory conditions. Spontaneous haemorrhage has been described in selected cases. Lymphadenopathy suggestive of nodal metastasis is uncommon, reported in only 0–12% of cases. These nonspecific clinical manifestations underscore the pivotal role of imaging and the vigilant radiologist in early detection and characterization. Ewing sarcoma does not have specific clinical features, but common manifestations include localized pain, stiffness, or swelling/mass that can last for weeks or months [12]. Depending on its location, Ewing sarcoma may also present as a pathological fracture [12]. Metastatic or primary spinal Ewing sarcoma of the spine may cause spinal cord compression [13]. Systemic symptoms such as fever and weight loss often indicate metastatic disease and can be associated with elevated serum levels of lactate dehydrogenase (LDH), leukocytes, and C-reactive protein (CRP) [14].

### Current Treatment Strategies

The standard treatment for localized Ewing sarcoma is multimodal, involving intensive multiagent chemotherapy combined with appropriate local treatment [15]. For patients under 18 years old, dose-dense chemotherapy regimens (every two weeks) using vincristine-doxorubicin-cyclophosphamide alternating with ifosfamide-etoposide (VDC-IE) have been shown to be more effective than regimens administered every three weeks [16]. The Euro Ewing 2012 trial further supports this, demonstrating the superiority of the VDC-IE regimen compared to a three-week interval vincristine-ifosfamide-doxorubicin-etoposide (VIDE) regimen [17].

Local treatment options include limb-sparing wide excision (or amputation) and/or definitive radiotherapy [15, 18]. Surgery is generally considered for patients where negative resection margins can be achieved while preserving reasonable functional outcomes [15]. Assistive (neo)adjuvant radiotherapy may be considered before or after surgery for patients with poor responses to induction chemotherapy, at risk for inadequate resections margins, or with (micro)residual tumors after resection [19, 20]. Definitive radiotherapy is an effective local control alternative to surgery when function-preserving surgery isn't feasible due to tumor location or extension, even after neoadjuvant chemotherapy, typically using doses in the 54–60 Gy range [21], though, in retrospective data from 1058 patients enrolled in CESS (Cooperative Ewing Sarcoma Study) 81, CESS 86, and EICESS (European Intergroup Cooperative Ewing Sarcoma Study) 92 trials, the local failure recurrence rates were significantly higher after definitive radiotherapy (26.3%) compared to surgery (7.5%) [18]. Modern technologies, such as stereotactic body radiotherapy (SBRT), intensity-modulated radiotherapy, and proton therapy [22], may offer potential improvements in local control and reduced toxicity [15]. Proton therapy is used to limit radiation dose and late effects in surrounding tissues, which is especially important for young children [23].

Local therapy treatment is also important for patients with metastatic Ewing sarcoma at diagnosis and should be considered in addition to systemic treatment whenever possible to improve event-free survival, since patients receiving local therapy treatment to both the primary and metastatic sites may have better event-free survival [24]. For patients with metastatic disease at diagnosis, systemic treatment is the same as for localized disease, and high-dose chemotherapy with stem-cell transplantation is not advisable except in clinical trials, as it does not seem to improve the prognosis [15]. For the treatment of pulmonary metastases, surgical excision may be considered for those patients with a limited number of nodules [25], and SBRT may also be an option [26]. Bilateral whole-lung irradiation seems to be recommended at the end of systemic treatment for good responder patients, with a boost to residual lung metastases [27, 28].

### Metastatic Pattern

Approximately 20% of patients may have metastatic disease at diagnosis [29], and subclinical metastasis could lurk even with apparent localized disease [15]. Older ages, tumor size larger than 8 cm, and pelvic lesions are considered risk factors for distant metastasis [30]. Analysis of the SEER database with 277 patients diagnosed with metastatic Ewing sarcoma at diagnosis (2010–2018), showed that the lung was the most common distant metastatic site (50%), followed by bone (40%), lymph node (7%), and liver (4%) [31]. For patients with metastatic Ewing sarcoma at diagnosis, three-year cancer-specific survival was 56.1% and bone metastases are an independent poor prognostic factor [31]. Similarly, a retrospective study of 91 patients with localized Ewing sarcoma at diagnosis reported that lung (50%) and bone (30%) were common sites of distant relapse after definitive treatment [32].

## Methodology

Although this is not a systematic review, most references were obtained using a search of the PubMed database with keywords “Ewing” or “Ewing’s” with and without “sarcoma” and a combination of keywords such as “epidemiology”, “imaging”, “radiology”, “radiography”, “computed tomography or CT”, “magnetic resonance imaging or MRI”, “nuclear medicine”, “positron emission tomography or PET”, “presentation”, “differential diagnosis”, “prognosis or prognostic”, “biopsy”, “staging”, “surveillance”, “treatment response”, “radiomics”, and “artificial intelligence or AI”. Additional references were obtained from the citations in these retrieved and reviewed articles. Although there were no strict inclusion or exclusion criteria, all articles were published in English.

## Imaging of Ewing Sarcoma

### Presenting Findings

Radiologically, Ewing sarcoma most commonly manifests as an aggressive skeletal lesion with a predominant intramedullary origin. In a large single-institution series of 126 patients treated between 2005 and 2019, intramedullary tumors accounted for 88.9% of cases, whereas extraskeletal and periosteal presentations were less frequent, representing 6.3% and 4.8% of cases, respectively [33]. Skeletal involvement predominantly affected flat bones (47.6%), with a marked predilection for the pelvis (26.2%) and, to a lesser extent, the scapula (6.3%) [33]. Long bones were involved in 42.1% of cases, most commonly the femur (18.3%), followed by the humerus (9.5%) and tibia (6.4%), while short bone involvement was rare, with the calcaneus affected in 3.9% of patients [33].

These findings are consistent with larger epidemiological and imaging series, which report a predilection for pelvis, extremities, and ribs (86%), particularly the femur (21%), tibia (8–11%), humerus (around 10%), and fibula (7–9%), ribs (8%), and sacrum (6%) [6]. When long bones are involved, lesions most frequently arise in the metadiaphyseal region, reported in 44–59% of cases, although epiphyseal extension may be observed in up to 10% of patients. Extraskeletal Ewing sarcoma, although uncommon in contemporary molecularly confirmed series, may account for up to 20% of cases and typically presents as a large, rapidly enlarging soft-tissue mass without overt bone involvement, potentially occurring in virtually any anatomic location [34]. These presenting findings are summarized in Table 1.

**Table 1:** Ewing sarcoma presenting findings

Site	Percentage of Cases
Intramedullary	88.9
Periosteal	4.8
Extraskeletal	6.3
**Bone**	
Flat	47.6
Pelvis	26.2
Scapula	6.3
Ribs	8
Mandible/Maxilla	1–2
Calvarium	1
Facial Bones	0.5
Sternum	0.2
Long	42.1
Femur	18–21
Humerus	10
Tibia	8–11
Fibula	7–9
Irregular	10–12
Sacrum	6
Spine	4–6
**Long Bone Region**	
Metadiaphyseal	44–59
Epiphyseal	10

### Conventional Radiography (CR)

Conventional radiography remains the first-line imaging modality in the evaluation of suspected Ewing sarcoma, although its findings, while often characteristic, are not pathognomonic [35]. In its most frequent intramedullary form, Ewing sarcoma typically presents as a large, aggressive-appearing lesion, with a reported at diagnosis (pre-therapeutic) median tumor size of approximately 9.9 cm (range: 2.4–21.0 cm), whereas periosteal Ewing sarcoma tends to be of comparable size, with a median diameter of 10.5 cm (range, 4.3–14.8 cm) [33]. The radiographic features described below primarily reflect the intramedullary form, which accounts for the vast majority of cases.

**Fig. 1:**
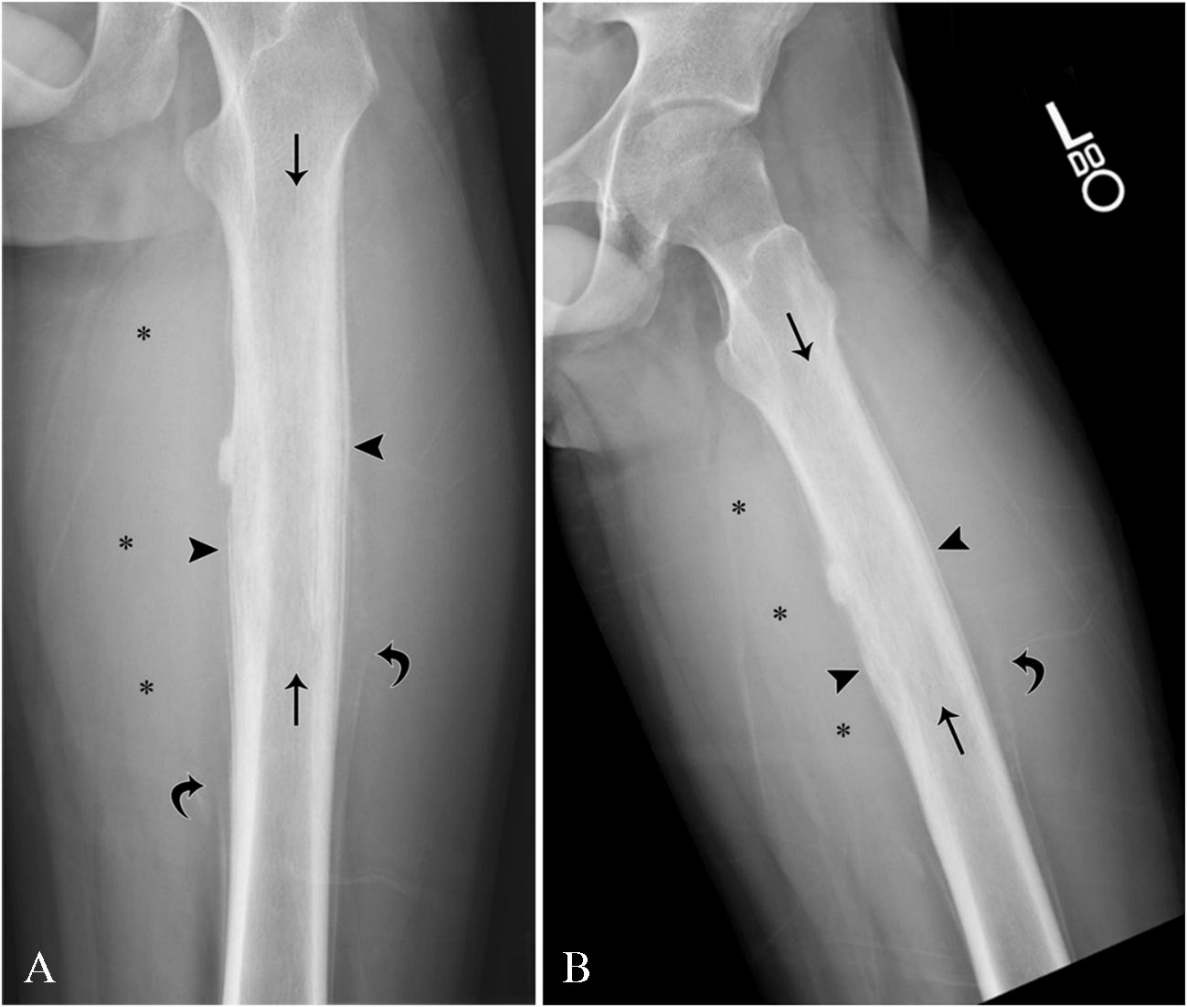
17-year-old male with Ewing sarcoma of the left femur. Anteroposterior (AP) (**A**) and lateral (**B**) radiographs demonstrate a permeative lytic lesion (arrows) with a combination of "onion peel" periosteal reaction (arrowheads) and Codman's triangle (curved arrows). A large extraosseous component can be extrapolated from marked adjacent soft tissue thickening (*)

**Fig. 2:**
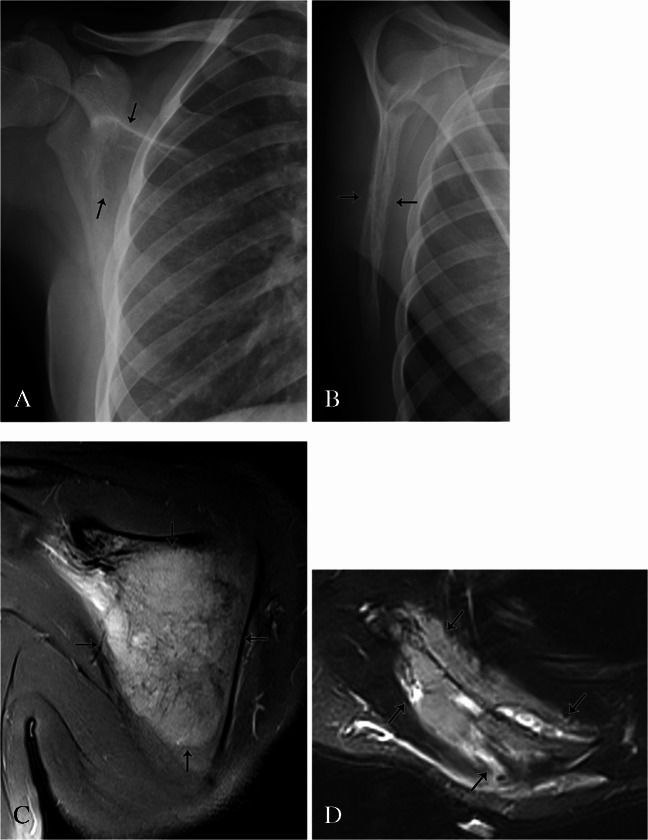
12-year-old female with Ewing sarcoma of the right scapula. AP (**A**) and lateral (**B**) radiographs demonstrate a permeative lytic lesion (arrows). Coronal (**C**) and axial (**D**) proton density (PD) fat saturation (FS) MRI images show significantly more extensive involvement than suggested on x-ray, as well as a considerable extra-osseous extension (arrows)

**Fig. 3:**
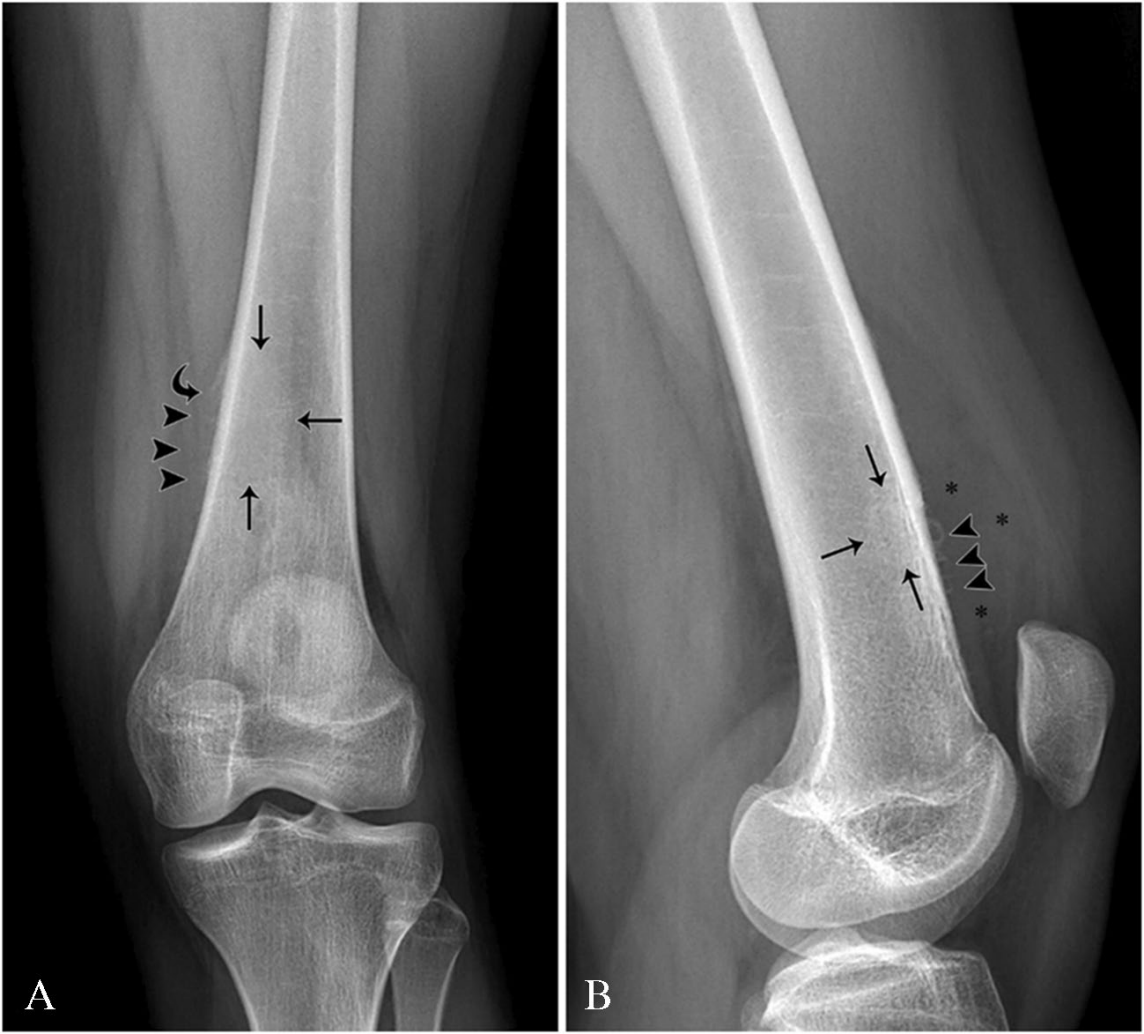
15-year-old female with Ewing sarcoma of the left femur. AP (**A**) and lateral (**B**) radiographs demonstrate a subtle mixed sclerotic and lucent lesion (arrows) with a medial Codman's triangle (curved arrow) and anterior "sunburst" periosteal reaction (arrowheads). Extra-osseous extension into the prefemoral space can be seen (*)

Typical features include the following [34]:

- **Zone of transition**: Wide zone of transition in ~ 96%. This includes Lodwick-Madewell type II (moth-eaten) and type III (permeative) lesions [36, 37].
- **Bone destruction pattern**: Permeative change is seen in 76–82% of patients. There may additionally be frank cortical breach or thinning without visible breach.
- **Location**: Metadiaphyses and diaphyses of long bones. While the diaphyseal location is often emphasized and can serve as a diagnostic clue, 44–59% of long bone lesions are metadiaphyseal. There is also a predilection for flat bones (approximately 40%), such as the iliac wings and scapulae.
- **Periosteal reaction**: The presence of visible periosteal reaction can be variable. The classic pattern of lamellated (so-called “onion-skin”) periosteal reaction is present in almost 60% of cases (Fig. 1) [38]. Other periosteal patterns, such as sunburst or Codman triangle, may also occur [39].
- **Soft‐tissue mass**: In the majority (approximately 96%) of patients, the tumor extends beyond osseous margins and into the adjacent soft tissues (Fig. 2). This, unfortunately, can be associated with a greater risk of metastasis [40]. When evaluating an aggressive bone lesion in an adolescent, it is thus imperative to look for an adjacent soft tissue mass.
- **Sclerosis/mixed lytic‐sclerotic pattern**: A mixed lytic-sclerotic appearance is typical (Fig. 3), while additional purely sclerotic lesions can occur [39].
- **Epiphyseal extension**: While not typically centered in the epiphyses, extension into the epiphyses may be present in up to 10% of patients. If this epiphyseal component becomes relatively large, it can impact the accuracy of pre-biopsy imaging interpretation.

**Fig. 4:**
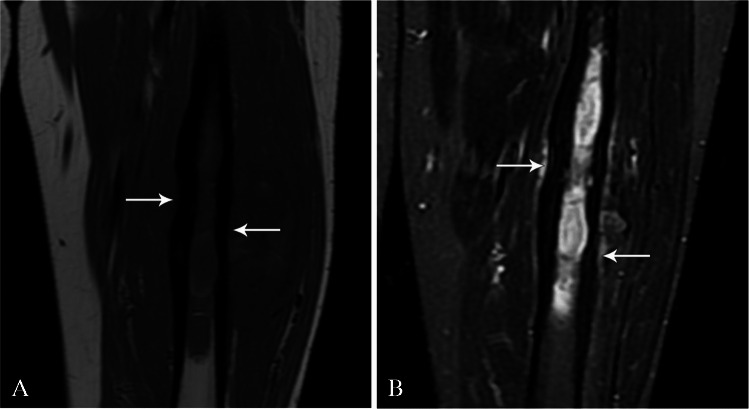
26-year-old male with Ewing sarcoma of the left mid femur. Coronal T1 (**A**) and STIR (**B**) MRI images demonstrate cortical thickening with saucerization defects (arrows) of both the medial and lateral aspects of the femoral shaft

Periosteal Ewing sarcoma represents a rare and distinct radiographic presentation. In a series of 11 patients, lesions were most commonly located in the proximal segments of long bones, without medullary cavity involvement and without metastatic disease at presentation [41]. These tumors often resemble other periosteal sarcomas, with the notable absence of matrix calcification serving as a potential discriminating feature. In a more recent series, periosteal lesions typically involved the diaphysis of long bones and demonstrated cortical-based growth with frequent septations, while saucerization of the outer cortical margin—classically described in periosteal tumors—was rather common, observed in 2/6 (33.3%) patients (Fig. 4) [33]. None of the periosteal lesions in this series demonstrated circumferential growth.

Ewing sarcoma involving the extremities of the hands and feet is rare, accounting for approximately 2–5% patients. In a historical cohort of 43 patients, lesions affecting the wrists, hands, feet, fingers, toes, and calcaneus shared the typical aggressive radiographic features of Ewing sarcoma, including permeative lytic destruction, aggressive periosteal reaction, cortical violation, and associated soft-tissue mass [42]. However, these tumors more frequently exhibited features suggestive of a less aggressive process, such as reactive bone formation, sclerotic components (observed in 42% of tumors), and bony expansion. Consequently, the radiographic appearance was classified as benign in 7% of cases, indeterminate in 35%, and overtly aggressive in 58%, highlighting the potential diagnostic pitfalls in this uncommon location.

### Computed Tomography (CT)

CT complements conventional radiography by improving the assessment of cortical integrity, periosteal reaction, and the osseous-soft tissue interface in both intramedullary and periosteal Ewing sarcoma. Overall, CT findings largely parallel those observed on radiographs, typically demonstrating aggressive cortical destruction associated with an adjacent soft-tissue mass in up to 95% of patients [43].

On CT, bone involvement most commonly exhibits a mixed or predominantly sclerotic appearance (approximately 68%), while purely lytic lesions account for about 32% patients [44]. Importantly, CT allows a more accurate distinction between sclerosis related to the tumor itself and sclerosis resulting from reactive periosteal bone formation, which may appear similar on radiograph [44]. Tumor-related osteoid or bone matrix production is not a histologic feature of Ewing sarcoma; therefore, sclerosing patterns observed on CT reflect reactive osteogenesis induced by contact with tumoral bone rather than true tumor bone formation [44].

CT is valuable for evaluating cortical breakthrough and the relationship between intraosseous and extraosseous components. Areas of focal cortical destruction with direct continuity between the medullary lesion and the soft-tissue mass are commonly identified. In addition, subtle linear low-attenuation cortical channels traversing an otherwise preserved cortex, allowing communication between the intraosseous and soft-tissue components, have been described in approximately 66% of cases (Fig. 4) [6, 45]. Conversely, in a minority of patients (about 17%), the cortex may appear intact on CT, with no visible communication between the intramedullary lesion and the extraosseous soft-tissue component [6, 45], a finding that may contribute to diagnostic uncertainty.

Following contrast administration, Ewing sarcoma demonstrates variable enhancement patterns on CT, reflecting heterogeneous tumor vascularity and necrosis, although CT remains limited in the characterization of intratumoral soft-tissue components compared with MRI [6, 45].

### Magnetic Resonance Imaging (MRI)

MRI is the optimal modality for evaluating Ewing sarcoma since it demonstrates the true lesion extension. The MRI protocol should cover the entire lesion as well as the entire bone where the lesion originates and the adjacent joints, since skip lesions can occur (14% of cases) [46, 47]. In addition to conventional MRI T1 and fluid-sensitive T2-weighted sequences, chemical shift and diffusion-weighted sequences with ADC (apparent diffusion coefficient) mapping can be performed. Ewing sarcoma lesions demonstrate low to intermediate T1 signal and variable low to intermediate (68%) or high T2 signal (32%) (Fig. 4) [6, 48, 49]. The areas of high T2 signal on fluid-sensitive sequences may be from hemorrhage, edema, or necrosis; therefore, the length of the tumor should be measured on the T1-weighted sequences since the fluid-sensitive fat-suppressed sequences may overestimate bone marrow involvement, as edema and marrow hyperplasia may show high T2 signal similar to that of tumor [6, 48, 50, 51].

**Fig. 5:**
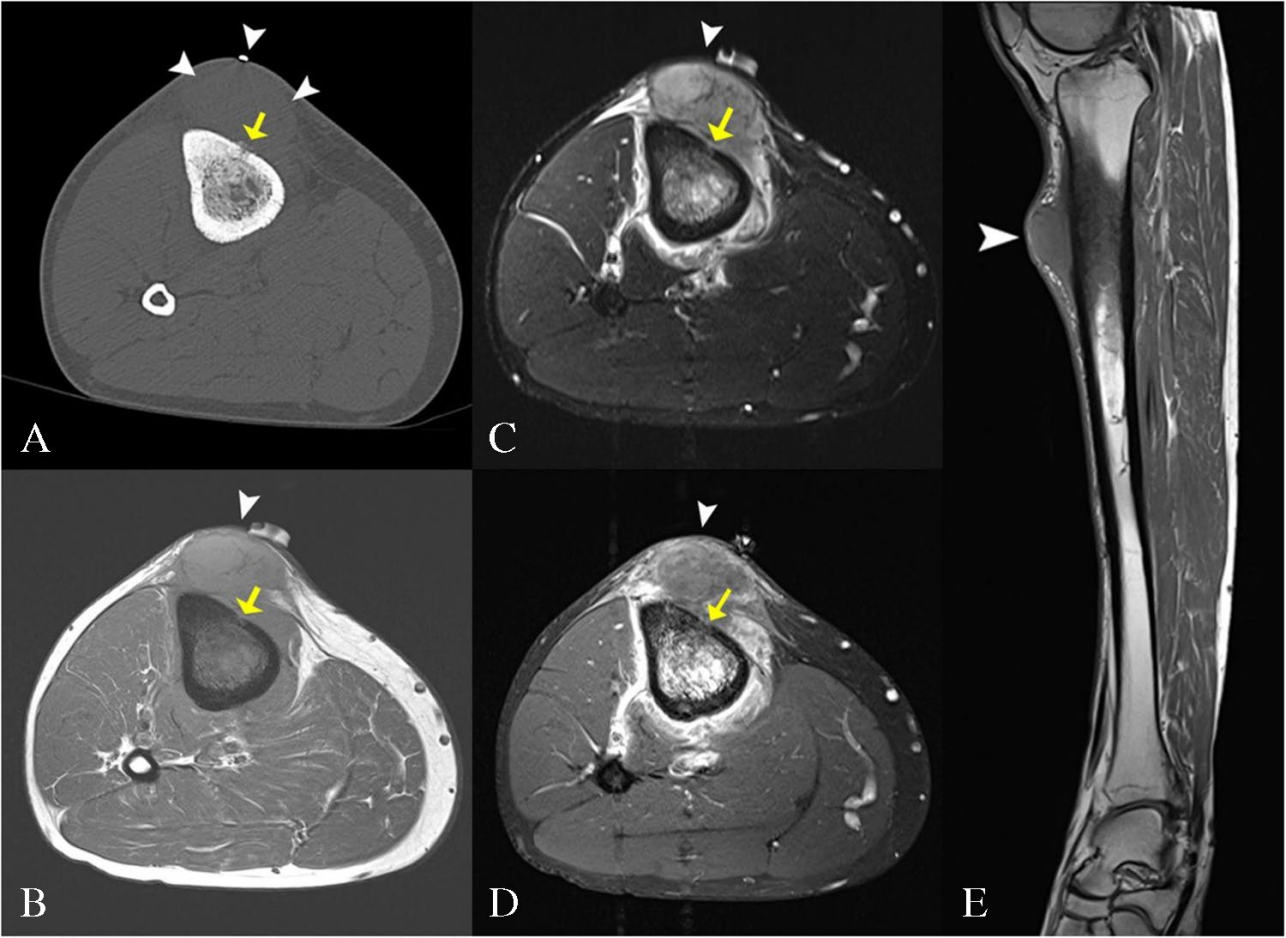
33-year-old male with Ewing sarcoma of the proximal tibia. Axial CT (**A**) and axial T1 (**B**), STIR (**C**), and T1 FS post contrast (**D**) and sagittal T1 (**E**) MRI images demonstrating low to intermediate T1 signal, variable intermediate to high T2 signal, and heterogeneous enhancement with a linear lower-attenuation/intermediate signal intensity channel extending through the low signal intensity cortex (arrows) allowing communication between the intraosseous and soft tissue components with an adjacent associated soft tissue mass (arrowheads)

**Fig. 6:**
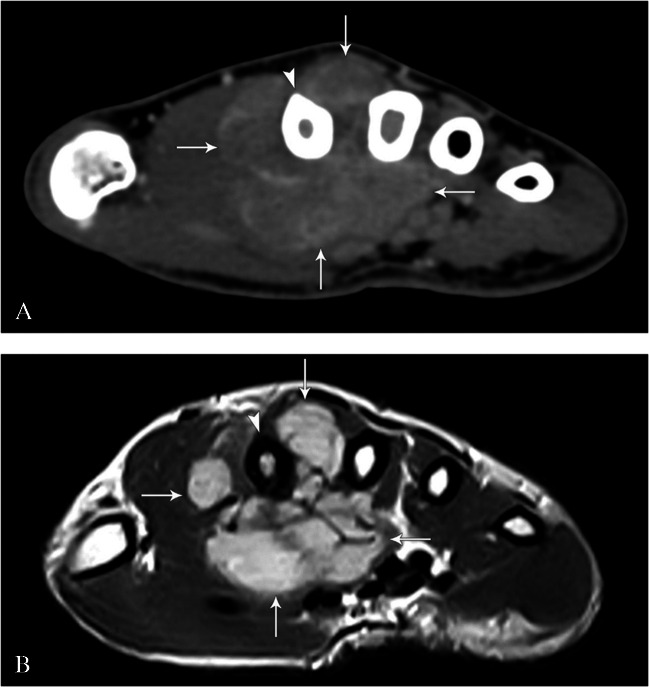
29-year-old male with Ewing sarcoma of the second metacarpal. Axial contrast-enhanced CT (**A**) and axial T2 (**B**) MRI images demonstrate a large extraskeletal mass (arrows) without any identifiable cortical disruption (arrowheads), also known as the wraparound sign

**Fig. 7:**
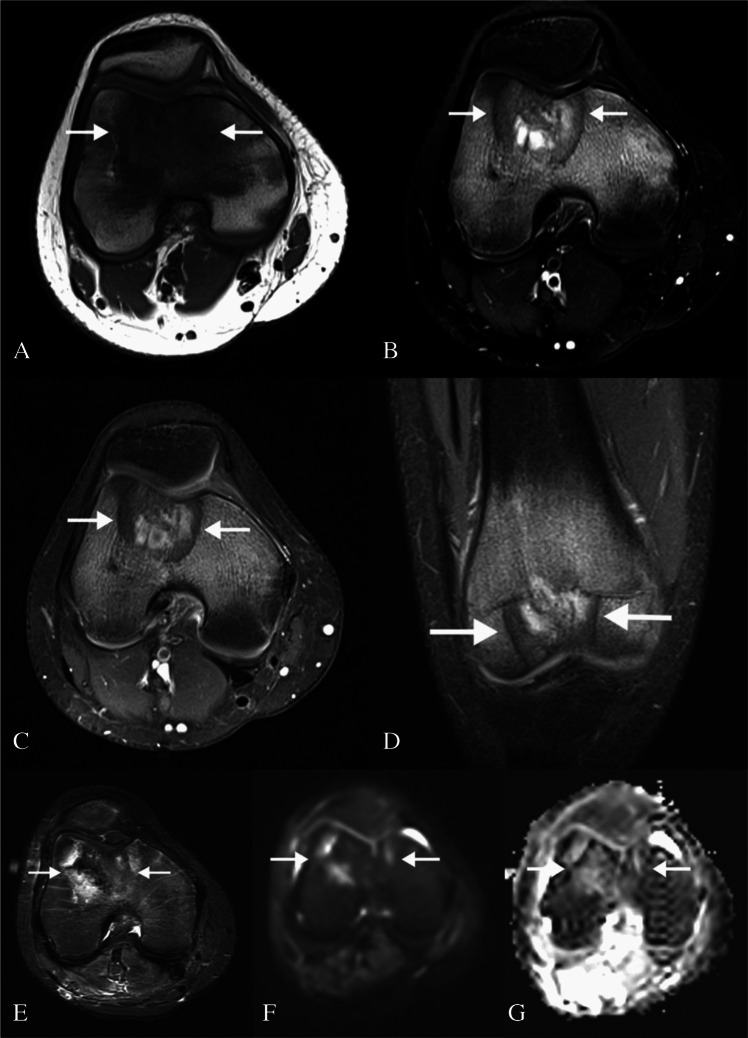
14-year-old female with Ewing sarcoma of the distal femoral epiphysis. Axial T1 (**A**) and STIR (**B**) and axial (**C**) and coronal (**D**) T1 FS post contrast MRI images demonstrate low to intermediate T1 signal, variable intermediate to high T2 signal, and heterogeneous enhancement with marked surrounding edema without an associated soft tissue mass. Axial STIR (**E**), DWI (**F**), and ADC map (**G**) images after radiation treatment demonstrate decreased T2 hyperintensity with few regions of T2 shine through but no restricted diffusion

The presence of a soft tissue mass on MRI is the most defining characteristic of Ewing sarcoma and is seen in the majority of cases [6, 46, 52]. Other characteristics include a sharp transition zone and focal areas of cortical destruction allowing for continuity between the medullary cavity and soft tissue component (92% of cases) [6]. Subtle linear intermediate signal intensity channels extending through the low signal intensity cortex can also be seen on MRI as seen on CT and may be the only evidence of cortical involvement and destruction on MRI (Fig. 5) [6]. Some cases may not demonstrate cortical disruption in which the cortex remains T2 hypointense, but there is a prominent soft tissue component that appears to be wrapped around the bone (wraparound sign), thought to be due to the permeative growth of the tumor through the Haversian canal system such that the calcified bone is not significantly destroyed (Fig. 6) [47]. Very few lesions do not demonstrate the presence of a soft tissue mass (Fig. 7).

Contrast enhancement is seen in all cases and is either diffuse or peripheral nodular (Fig. 5 and Fig. 7) [6]. Dynamic contrast-enhanced (DCE)-MRI may differentiate areas of necrosis, viable tumor, fibrosis, and peritumoral edema, since they show specific types of time intensity curves (TIC) [50]. Although there may be overlap, DCE-MRI can help differentiate or narrow the differential diagnosis between a benign tumor (no uptake or progressive contrast uptake), malignant tumor (delayed plateau or plateau with delayed washout), or fibrosis or granulation tissue (fast initial contrast uptake followed by progressive late enhancement) [50].

Diffusion-weighted imaging (DWI) shows a restriction pattern with very low apparent diffusion coefficient (ADC) values that can help differentiate edema or necrosis from tumor cellularity. These images are routinely performed in the axial plane. DWI imaging is based on measuring the random Brownian motion of water molecules within tissue, and the ADC is a quantitative measure of this movement in which low ADC values in tumors reflect areas where there is limited diffusion from an abundance of cell membranes, and high ADC values are observed in acellular regions such as in edema, necrosis, or benign neoplasms [53, 54]. Ewing sarcoma is a highly cellular tumor and usually shows restricted diffusion, which correlates with low ADC values [50, 55]. Absolute ADCmin has also been shown to differentiate Ewing sarcoma from osteosarcoma, with ADCmin values in Ewing sarcoma (0.566 ± 0.07 × 10^–3^ mm^2^/s) reported to be significantly lower than in osteosarcoma (1.193 ± 0.33 × 10^–3^ mm^2^/s) [56]. Diffusion-weighted imaging is also helpful in assessing tumor treatment-related response (Fig. 7).

**Fig. 8:**
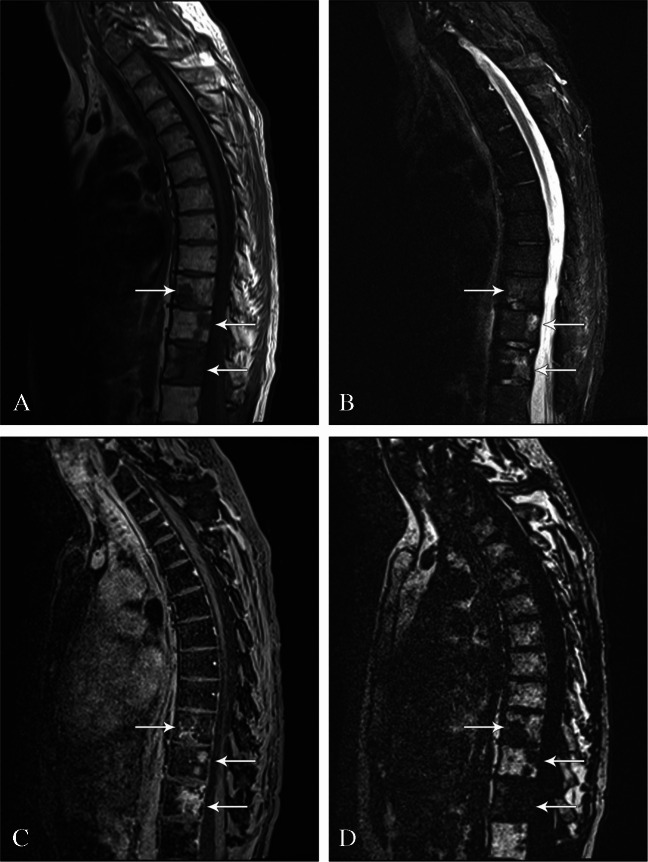
38-year-old male with metastatic Ewing sarcoma. Sagittal T1 (**A**) and STIR (**B**) and T1 Dixon volumetric interpolated breath-hold examination (VIBE) post-contrast Water (**C**) and Fat (**D**) MRI images demonstrate metastases of the T10-12 vertebra (arrows) that enhance and replace the normal fatty bone marrow

In-phase and out-of-phase chemical shift MRI can differentiate between non-neoplastic and neoplastic marrow lesions by detecting microscopic fat (Fig. 8). Malignant tumors, including Ewing sarcoma, and non-fat-containing benign bone tumors do not demonstrate signal dropout on the out-of-phase images [57, 58]. Red marrow and most non-neoplastic processes, such as fractures, edema, marrow hyperplasia, ischemia, and infection, do not fully replace bone marrow fat and preserve some degree of signal cancellation or dropout on the out-of-phase images [57–59]. Chemical shift imaging (CSI) is useful in differentiating indeterminate marrow lesions; however, the presence of a soft tissue mass, cortical destruction or disruption, and periosteal reaction in most Ewing sarcoma cases usually allows a confident diagnosis to be made.

Whole-body (WB)-MRI also has a high sensitivity, specificity, and diagnostic accuracy for the identification of metastases in Ewing sarcoma and is more sensitive than bone scintigraphy in detecting skeletal metastases, with the exception of skull vault metastases [60, 61]. The protocol usually includes coronal T1, coronal STIR (short tau inversion recovery), and axial DWI with ADC mapping [62]. Gradient echo-based Dixon sequences can be performed as an alternative to conventional spin-echo sequences with four imaging types generated with one Dixon acquisition (in-phase (IP), out-of-phase (OP), fat-only, and water-only images), with fat-only images providing the greatest contrast for focal lesions when compared with background marrow (Fig. 8) [62, 63]. WB-MRI using T2-Dixon fat and water reconstructions has shown similar accuracy to T1WI and STIR combination in the evaluation of skeletal metastases in patients with primary solid cancers with significantly shorter acquisition time [64, 65].

### Nuclear Imaging and Positron Emission Tomography (PET)

**Fig. 9:**
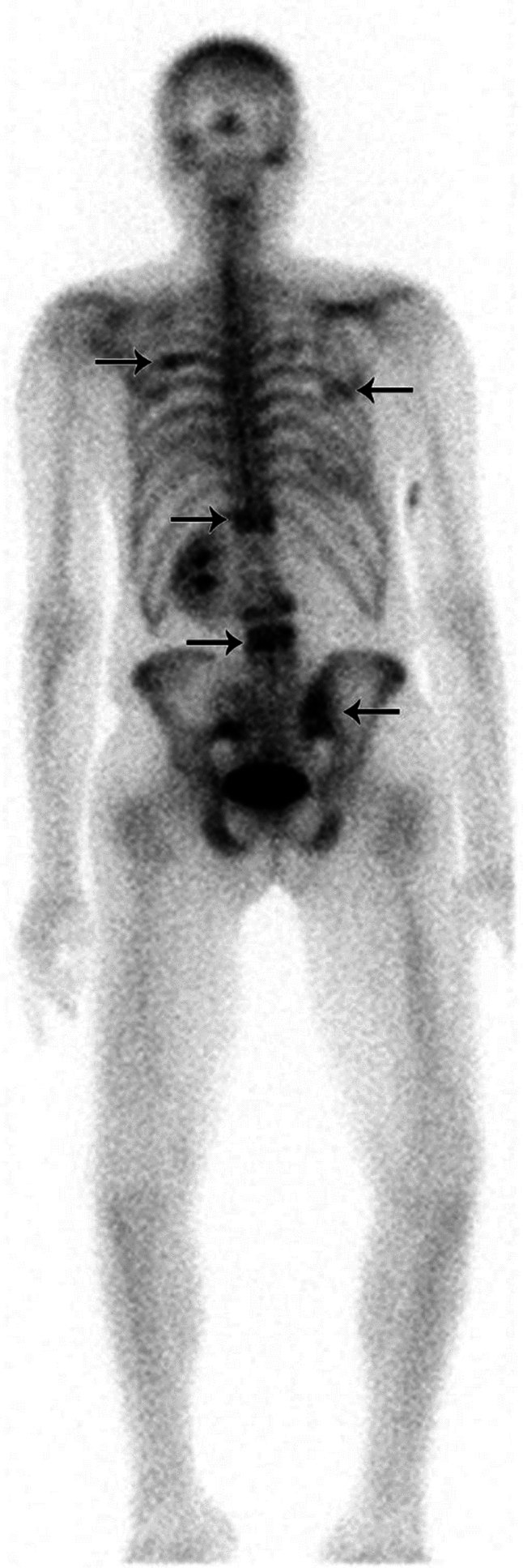
38-year-old male with Ewing sarcoma of the left kidney. Tc99m-MDP bone scan demonstrates multifocal tracer uptake of ribs, vertebra, and the left ilium, corresponding to osseous metastases. Note the surgically absent left kidney

**Fig. 10:**
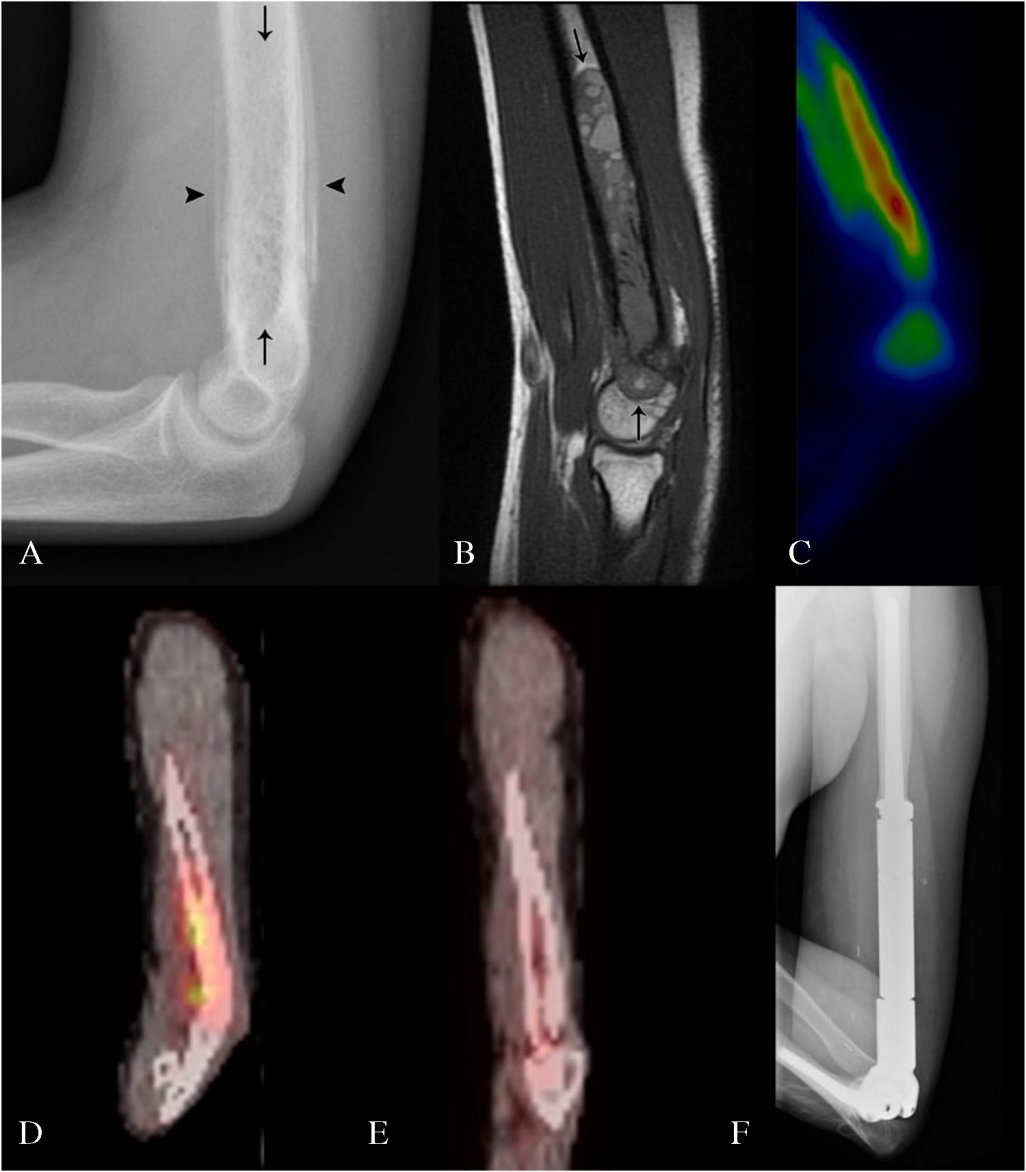
17-year-old male with Ewing sarcoma of the distal humerus. Lateral radiograph (**A**) demonstrates a permeative lytic lesion (arrows) with periosteal reaction (arrowheads). Sagittal T1 MRI (**B**) better shows the full extent of the lesion (arrows). Sagittal reformation of SPECT-CT Tc99m-MDP bone scan (**C**) displays intense tracer uptake within the lesion. Pre-treatment sagittal reformation of 18F-FDG PET-CT (**D**) reveals intense metabolic activity with an SUVmax of 4.8. After completing 9 weeks of chemotherapy with vincristine, doxorubicin and cyclophosphamide, sagittal reformation of 18F-FDG PET-CT (**E**) demonstrates resolution of the previously seen metabolic activity, indicating excellent treatment response. The patient subsequently underwent limb salvage surgery with wide resection, with a lateral radiograph (**F**) showing modular endoprosthesis placement

Nuclear medicine can play an important role in the evaluation of Ewing sarcoma. Technetium-99 m (^99m^Tc)-labeled methylene diphosphonate (MDP) bone scintigraphy demonstrates uptake within Ewing sarcoma on blood flow, blood pool, and delayed images, although there are rare reports of photopenia [6]. Historically, ^99m^Tc- MDP bone scintigraphy has been used to detect skeletal metastases (Fig. 9). In a study of 72 patients with Ewing sarcoma, bone scan demonstrated unsuspected metastases in 28 patients (39%), including additional unsuspected metastases in 10 patients with clinically suspected or radiographically demonstrated metastases [66]. Primary disease in the axial skeleton (71%) is more frequently associated with osseous metastases than primary disease of the upper (41%) or lower (45%) extremities [66]. Initial bone scan activity within the primary Ewing sarcoma lesion does not have prognostic significance for predicting long-term survival or disease progression [67]. However, after treatment, a bone scan that becomes and remains negative indicates no local recurrence, whereas if activity remains positive or becomes negative but later shows increased uptake, there is a high risk for local recurrence, although pathological fracture or heterotopic ossification may also present this way [68]. Single-photon emission computed tomography (SPECT), especially when combined with CT, can better localize tracer uptake (Fig. 10).

Gallium-67 (^67^ Ga) citrate scintigraphy also shows uptake in most cases of primary Ewing sarcoma but is less sensitive for the detection of osseous metastases [69, 70]. However, ^67^ Ga-citrate activity is more specific than ^99m^Tc-MDP uptake for evaluation of post-treatment recurrence [67].

**Fig. 11:**
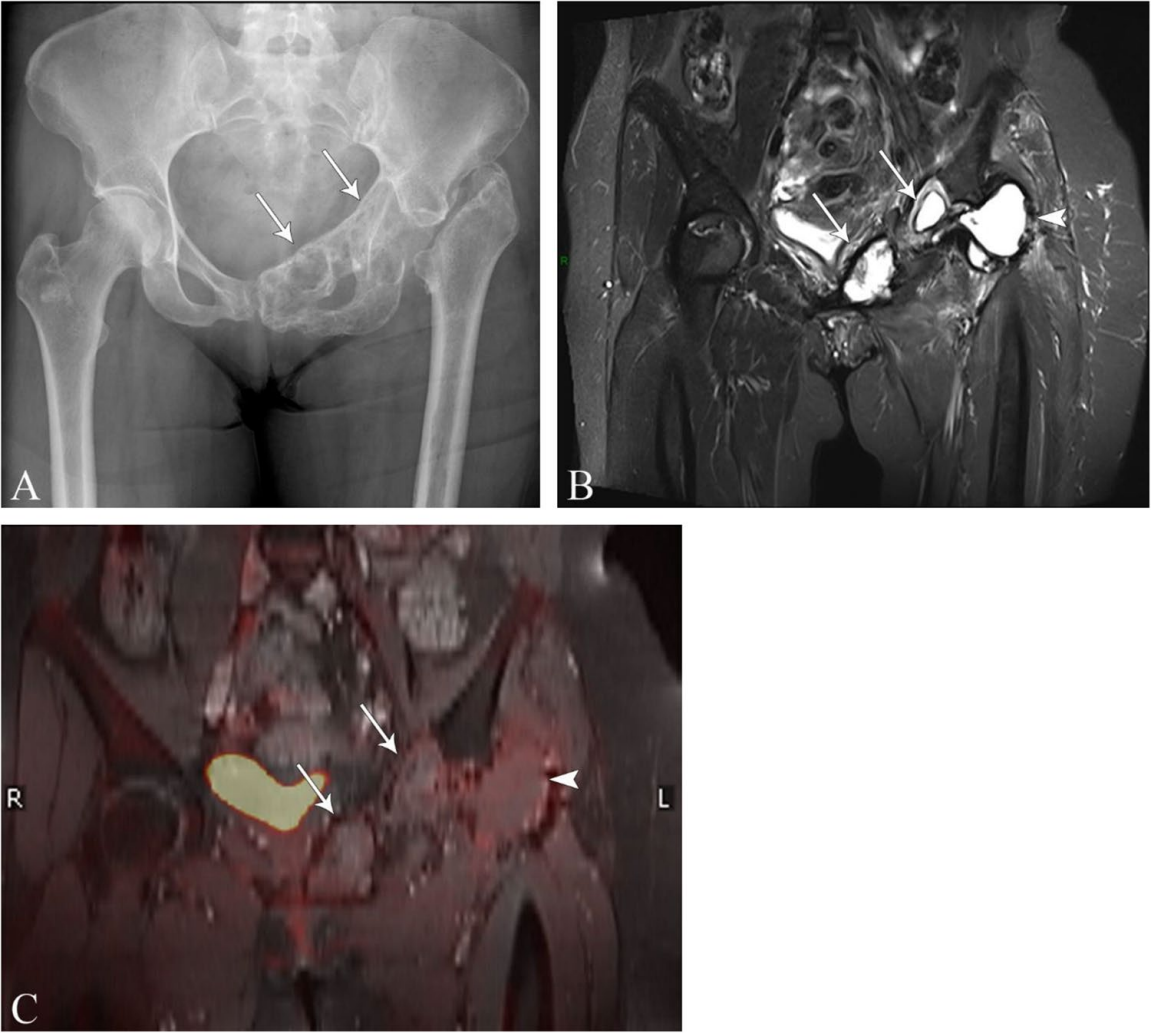
44-year-old female with pelvic Ewing sarcoma status post chemoradiation therapy. AP radiograph (**A**), coronal STIR MRI image (**B**) and coronal fused ^18^F-FDG PET-MRI image (**C**) demonstrate lytic/hyperintense left acetabular and superior pubic rami lesions (arrows) and small volume of fluid at the Girdlestone pseudoarthrosis (arrowheads) without significant metabolic activity, confirming treated disease

2-[fluorine-18] (^18^F) fluoro-2-deoxy-D-glucose (FDG) positron emission tomography (PET) also demonstrate activity within Ewing sarcoma due to the increased rate of glycolysis in these tumors [71]. ^18^F-FDG PET and particularly hybrid PET/CT can determine the presence (100% sensitivity) and extent of primary Ewing sarcoma and detect lymph node (90% sensitivity) and osseous metastases (90% sensitivity) [72]. ^18^F-FDG PET/CT is superior to bone scan for the detection of osseous metastases, detecting 18–25% more lesions [73, 74]. However, when Ewing sarcoma is sclerotic, bone scintigraphy may detect osseous metastases not detected by ^18^F-FDG PET/CT [75]. ^18^F-FDG PET may also allow the estimation of histologic grade and to predict patient outcomes such as overall survival, both before and after neoadjuvant therapy, by measuring the standardized uptake value (SUV) [76, 77]. The mean SUV value for Ewing sarcoma is 5.3 [78]. The main downside of ^18^F-FDG PET is the lack of specificity, as metabolically active inflammatory and infectious processes and some benign tumors can also show uptake [79]. As this includes postoperative and posttraumatic inflammation and granulation tissue, postoperative imaging should be performed at least 3 months after surgery. The high sensitivity of ^18^F-FDG PET has obviated the need for bone marrow biopsy/aspiration as a routine part of the initial staging for Ewing sarcoma [80, 81]. Compared to conventional imaging (CT, MRI, and bone scintigraphy), ^18^F-FDG PET/CT showed improved overall diagnostic performance for staging and follow-up compared to both CT and bone scan and similar performance compared to MRI [82]. However, CT is more reliable than ^18^F-FDG PET for the detection of small pulmonary metastases (< 7 mm) [71, 81]. Post-treatment ^18^F-FDG PET/CT is highly sensitive and accurate in detecting recurrence and metastases of Ewing sarcoma (93.7% sensitivity, 87.5% specificity, and 91.7% accuracy) and predicts progression-free survival (Fig. 10) [83]. Hybrid ^18^F-FDG PET/MRI combines the benefits of both modalities for the evaluation of Ewing sarcoma, with less radiation dose compared with PET/CT, especially important in the pediatric population (Fig. 11) [84].

PET/CT has shown concordance with systematic iliac crest biopsy in 19 of 20 patients, with one additional lesion detected by PET/CT [85]. In mixed sarcoma cohorts, including osteosarcoma and Ewing sarcoma, PET and WB-MRI were concordant for tumor length and skip metastases in 91.5% of lesions; however, WB-DWI MRI depicted more than twice as many malignant bone or marrow sites (n = 151 vs 60) and lymph node sites (n = 59 vs 28) as PET/CT (p < 0.001) [86]. Overall, ^18^F-FDG PET/CT seems superior for metabolic characterization and whole-body staging, whereas WB-MRI with DWI offers greater sensitivity for marrow and skeletal involvement, emphasizing the complementary role of these modalities in comprehensive Ewing sarcoma evaluation.

## Main differential diagnosis and discriminative features

The main differential diagnoses of Ewing sarcoma include acute osteomyelitis, osteosarcoma, and lymphoma and, particularly in soft tissues, other genetically defined round cell sarcomas such as BCOR (BCL6 Corepressor)- and CIC (Capicua Transcriptional Repressor)- rearranged sarcomas [87–90].

(i) *Ewing Sarcoma versus Osteomyelitis*: distinguishing Ewing sarcoma from acute osteomyelitis remains a well-recognized diagnostic challenge, especially in children and adolescents presenting with pain, fever, and elevated inflammatory markers. In a cohort of 28 patients with diagnostic uncertainty, Henninger et al. identified several highly discriminative imaging features [88]. The most decisive criterion was the presence of a sharper and better-defined margin of the bone lesion, observed in all patients with Ewing Sarcoma (p<0.0001). Contrast-enhancing soft-tissue masses and cortical destruction were present in all Ewing sarcoma patients, whereas four patients with osteomyelitis showed no cortical reaction (p=0.0103). Cystic or necrotic areas were significantly more frequent in Ewing sarcoma than in osteomyelitis (13 patients vs 1 patient; p=0.004) [88].

McCarville et al. further evaluated radiographic and MRI features differentiating Ewing sarcoma from osteomyelitis [89]. On radiography, joint or metaphyseal involvement, a wide transition zone, Codman triangle, periosteal reaction, and the presence of a soft-tissue mass were each significantly more common in Ewing sarcoma (p≤0.05). On MRI, permeative cortical involvement and a soft-tissue mass favored Ewing sarcoma (p≤0.02), whereas a serpiginous tract was more suggestive of osteomyelitis (p=0.04). Demographically, African American patients were more likely to have osteomyelitis than Ewing sarcoma (p<0.0001). Multivariate analysis identified only ethnicity and the presence of a soft-tissue mass as independent predictors (p≤0.01). Biopsy performance also differed, with diagnostic yields of 100% for open biopsy and 58% for percutaneous biopsy in osteomyelitis, compared with 88% and 50%, respectively, in Ewing sarcoma [89].

(ii) *Ewing Sarcoma versus Osteosarcoma*: Ewing sarcoma generally affects younger patients, with a peak incidence in the first and second decades of life, whereas osteosarcoma more often presents during adolescence and early adulthood [91]. Clinically, systemic symptoms such as fever and elevated inflammatory markers are more frequently associated with Ewing sarcoma and are uncommon in osteosarcoma. On radiographs and CT, Ewing sarcoma typically demonstrates permeative bone destruction, a wide zone of transition, minimal tumor matrix, and a disproportionately large soft-tissue mass, while osteosarcoma characteristically shows aggressive osteoid matrix mineralization [91]. On MRI, Ewing sarcoma more often exhibits relatively homogeneous marrow replacement, extensive extraosseous extension, and lower ADC values reflecting high cellularity, whereas osteosarcoma tends to display greater intratumoral heterogeneity with mineralized components and higher ADC values.

(iii) *Ewing Sarcoma versus BCOR- and CIC-rearranged Sarcomas*: BCOR- and CIC-rearranged sarcomas represent newly defined entities within the spectrum of round cell sarcomas, particularly in soft tissues, and may closely mimic Ewing sarcoma radiologically, especially extraskeletal Ewing sarcoma [6, 90]. These tumors typically demonstrate intermediate signal intensity on T1-WI, high signal intensity on T2-WI, and heterogeneous but intense contrast enhancement. In osseous BCOR sarcoma, marked signal heterogeneity has been suggested as a key differentiating feature compared with Ewing sarcoma, which appears homogeneous in approximately 86% of patients due to its high cellularity, with T2-weighted heterogeneity reported in only 14% of cases. Calcifications may provide an additional clue, as soft-tissue calcifications were observed in 40% of BCOR sarcomas reported by Brady et al., compared with only 7–9% of skeletal Ewing sarcomas [90].

## Role and Technique of Image-Guided Biopsy

**Fig. 12:**
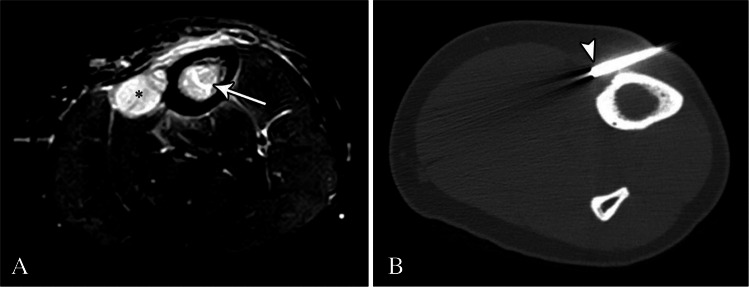
20-year-old male presented with a progressive, painful lump in the anterior aspect of the left leg. MRI. (**A**) T2 fat saturation axial image reveals an aggressive lesion of the tibial diaphysis (arrow) with a large extra-skeletal mass (asterisk). Orthopaedic oncologist submitted the patient to a CT-guided biopsy. (**B**) performed with an 8-gauge trap system needle (arrowhead). The final histopathological diagnosis was Ewing Sarcoma

Although open surgical biopsy was historically considered the reference standard for diagnosing Ewing sarcoma, image-guided core needle biopsy (CNB) has become the preferred initial approach in expert centers (Fig. 12). Earlier series reported variable diagnostic yields ranging from 50 to 88% in small cohorts of 5–40 patients. More recent data demonstrate significantly improved performance. In a large series of 139 patients with osseous and soft-tissue Ewing sarcoma undergoing 141 image-guided biopsy procedures, a definitive diagnosis was achieved in 97.1% of cases, with only three repeat biopsies required and a single immediate complication reported [92]. CT guidance is preferred for osseous lesions, and applicable to almost all the skeletal segments, whereas ultrasound guidance is suitable for superficial soft-tissue masses or appendicular skeletal lesions with cortex disruption [93, 94]. Biopsies should be performed using a coaxial technique in specialized sarcoma centers, with careful planning of the needle tract to minimize contamination of uninvolved tissues and to allow inclusion of the tract within the definitive surgical resection; skin marking or tattooing of the biopsy site may facilitate subsequent identification after neoadjuvant chemotherapy.

## Disease Staging

Disease staging is a critical determinant of prognosis and management in Ewing sarcoma. The conceptual framework for musculoskeletal tumor staging was introduced by Enneking in 1980 to integrate the most relevant prognostic factors related to local tumor extent, risk of recurrence and metastasis, surgical planning, and the need for adjuvant therapies [95, 96]. Imaging is central to each step of this process.

Conventional radiography is the first-line examination for suspected Ewing sarcoma. Definitive local staging relies on MRI of the primary tumor to accurately delineate intramedullary extent, cortical destruction, extraosseous extension, joint involvement, and skip lesions. Chest CT is mandatory to assess pulmonary metastases. Whole-body imaging is required for distant staging. Historically, this included skeletal survey and/or Technetium-99 m MDP bone scintigraphy; however, skeletal surveys alone are now considered inadequate because of their low sensitivity, particularly for early or intramedullary disease [97]. Whole-body MRI has demonstrated higher sensitivity than both skeletal survey and bone scintigraphy for detecting skeletal metastases, although its routine use may be limited by availability and reimbursement issues. Considering these issues, the Children’s Oncology Group (COG) recommends FDG PET/CT at initial staging [60, 97].

Local disease classification has traditionally relied on the Musculoskeletal Tumor Society (MSTS) staging system derived from the Enneking classification, which incorporates tumor grade (low vs high), anatomic extent (intra- vs extracompartmental), and the presence or absence of metastases (Table 2) [95, 96, 98–101]. The American Joint Committee on Cancer (AJCC) staging system further integrates tumor size and distinguishes between pulmonary, nodal, osseous, and other distant metastases, and is widely adopted in oncologic practice (Table 3) [100].

**Table 2:** Musculoskeletal Tumor Society (MSTS)/Enneking Staging System

Stage	Grade	Site	Metastases
IA	Low	Intracompartmental	None
IB	Low	Extracompartmental	None
IIA	High	Intracompartmental	None
IIB	High	Extracompartmental	None
III	Any	Any	Present

**Table 3:** American Joint Committee on Cancer (AJCC) Staging System

Stage	Tumor	Nodes	Metastases	Grade	Summary
IA	T1	N0	M0	G1 or GX	T1 low grade
IB	T2 or T3	N0	M0	G1 or GX	T2 or T2 low grade
IIA	T1	N0	M0	G2 or G3	T1 high grade
IIB	T2	N0	M0	G2 or G3	T2 high grade
III	T3	N0	M0	G2 or G3	T3 high grade
IVA	Any	N0	M1a	Any	Lung metastases
IVB	Any	N1	Any	Any	Regional lymph node involvement
	Any	Any	M1b	Any	Bone or other distant metastases

T1 ≤ 8 cm; T2 > 8 cm; T3 = “skip lesions”

G1 = well differentiated (low grade); G2 = moderately differentiated (intermediate grade); G3 = poorly differentiated (high grade); GX = cannot be assessed

N0 = no regional lymph node metastasis; N1 = regional lymph node metastasis

M0 = no distant metastasis; M1 = distant metastasis; a = lung; b = bone or other

The presence of metastatic disease at diagnosis represents the most significant adverse prognostic factor in Ewing sarcoma [2, 17]. Metastatic disease is reported in approximately 26% of patients at presentation, involving the lungs in 10%, bone or bone marrow in 10%, and other sites in 6% [4]. Across series, 15–46% of patients may present with gross metastatic disease, reducing five-year survival from approximately 35–71% to 0–34% [2, 4, 7, 12, 13, 15].

Among patients with metastatic disease, the lungs are the most common site (up to 80%), followed by bone (approximately 40%). Osseous metastases are consistently associated with a poorer prognosis compared with isolated pulmonary metastases [3, 16, 17]. In bone Ewing sarcoma, five-year overall survival is approximately 65.2%, increasing to 78.6% in localized disease, but decreasing to 40.1% in patients with isolated pulmonary metastases and to 28.1% in those with extrapulmonary metastases [12]. With current multimodality treatment combining chemotherapy, surgery, and radiotherapy, five-year survival is 60–70% for localized disease but only 20–40% for metastatic disease [4]. Less common metastatic sites include the brain or spinal cord, involved in up to 20% of patients with metastases, whereas soft-tissue and peritoneal involvement occur in fewer than 10% of cases; dural involvement is rare.

Traditionally, a bone marrow biopsy (BMB) is a crucial component of staging Ewing sarcoma to check for cancer spread [102]. However, because advanced imaging techniques like FDG-PET/CT and whole-body MRI are so sensitive at detecting bone marrow metastases, they may eventually replace BMB in order to reduce the need for invasive procedures [85, 102].

## Prognostic Imaging Features

Several imaging-derived features are useful for prognostic stratification in patients with Ewing sarcoma [103, 104]. As in most solid malignancies, including musculoskeletal sarcomas, tumor size on baseline imaging is a key prognostic determinant [105]. A systematic review and meta-analysis identified tumor dimensions among the most important prognostic factors in Ewing sarcoma, with larger tumors being associated with poorer survival outcomes [106]. Specifically, a longest tumor diameter greater than 8 cm and a tumor volume exceeding 200 mL were significantly associated with reduced event-free survival [106]. In Ewing sarcoma, tumor volume may be a more appropriate metric than maximal diameter, even when estimated using ellipsoid or cylindrical formulas, because involvement of flat bones and/or the presence of a large extraosseous soft-tissue component may render a single linear measurement less representative of true tumor burden [107, 108].

Tumor location is another well-established prognostic factor. Primary tumors arising in surgically challenging sites, particularly the axial skeleton and pelvis, are associated with worse outcomes. Patients with pelvic Ewing sarcoma have significantly poorer overall survival compared with those with tumors located in the extremities. In contrast, extraskeletal Ewing sarcoma has been associated with a more favorable prognosis, with a modest but statistically significant improvement in 5-year survival compared with skeletal Ewing sarcoma (69.7% vs 62.6%, p = 0.02) in an analysis of more than 2,000 patients [109]. In skeletal Ewing sarcoma, the presence and extent of the associated soft-tissue mass may further influence prognosis; infiltration of adjacent structures, such as lung parenchyma in rib lesions, has been linked to more aggressive tumor biology and an increased risk of relapse [110].

**Fig. 13:**
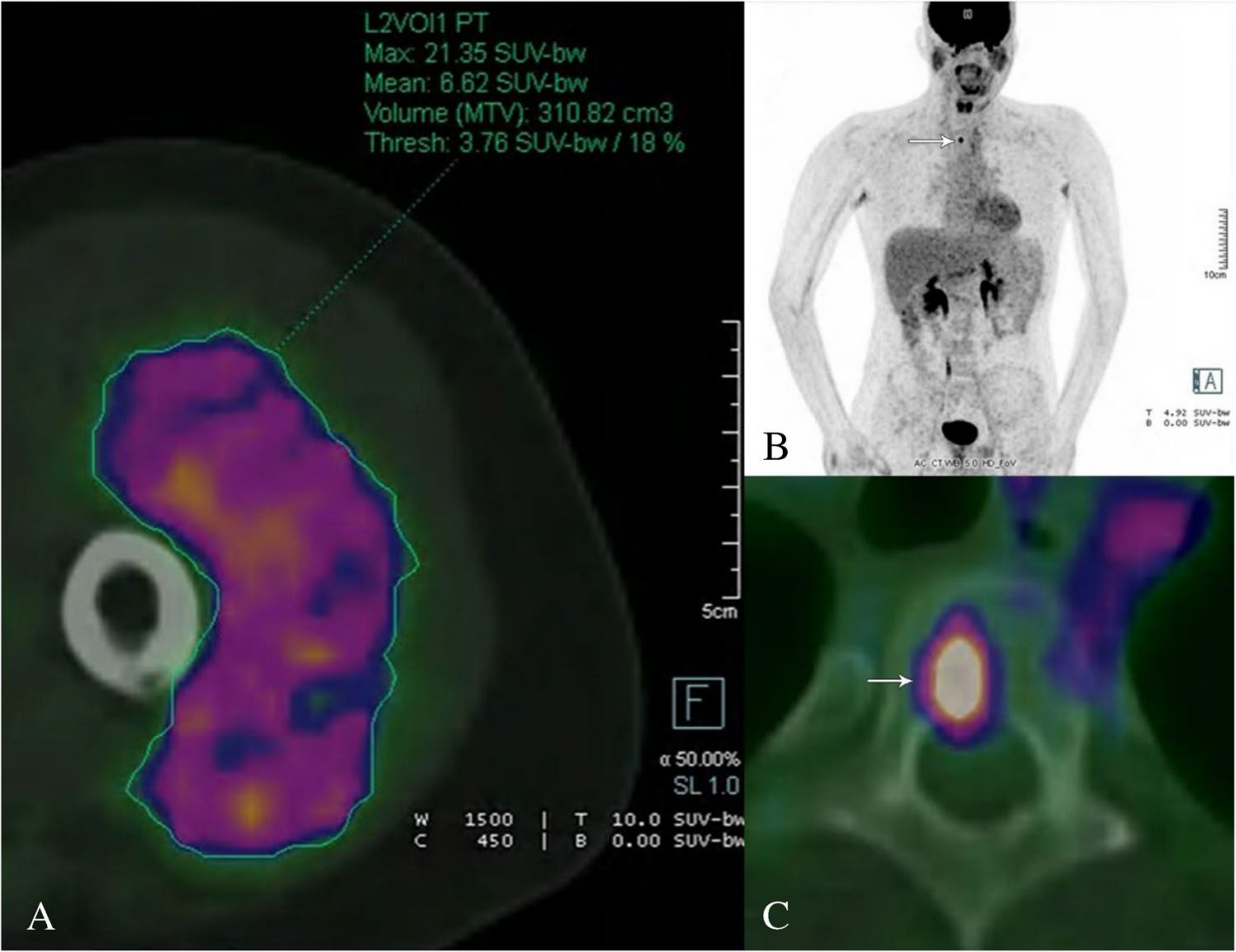
20-year-old male with Ewing sarcoma of the left femur diaphysis with a large extraskeletal component. Baseline axial ^18^F-FDG PET-CT (**A)** showed large dimensions (max diameter 11 cm, vol. > 200 ml), high SUV (max 21, mean 6.6). The patient presented with a single skeletal metastasis at diagnosis of the T3 thoracic vertebral body on whole-body MIP (**B**) and axial PET-CT (**C**) (arrows)

^18^F-FDG PET imaging also plays an important role in prognostic stratification. Multiple studies have demonstrated that higher SUV values at baseline and after induction chemotherapy are associated with worse outcomes (Fig. 13). Salem et al., in a cohort of 28 patients, reported that a baseline SUV > 11.6 was associated with inferior overall survival (hazard ratio [HR] = 5.71; 95%CI: 1.85–17.61; p = 0.003) and progression-free survival (HR = 3.16; 95%CI: 1.13–8.79; p = 0.03) [111]. Similarly, Palmerini et al. found that a SUVmax > 6.1 in 45 patients was associated with reduced 3-year event-free survival (p = 0.004) [112]. Hwang et al., in a study of 34 patients, reported significantly shorter survival in patients with SUVmax > 5.8 compared with those with lower values (656 vs 1,265 days; p = 0.002) [113]. Notably, each unit increase in baseline SUVmax was associated with a 5% increase in the hazard of death (HR = 1.05; 95%CI: 1.0–1.1; p = 0.01), while after induction chemotherapy, each unit increase in SUVmax was associated with a higher hazard ratio of 1.2 (95%CI: 1.0–1.4; p = 0.01).

Finally, the presence of distant metastases at diagnosis, as detected by imaging, remains one of the strongest adverse prognostic factors in Ewing sarcoma, with reported hazard ratios for reduced overall survival ranging from 2.4 to 4.4 in high-quality cohorts [106]. Nevertheless, responsiveness to chemotherapy remains a critical determinant of outcome, and all imaging markers of treatment response, including changes in metabolic activity on PET, have consistently been associated with prognosis. These aspects are discussed in detail in the subsequent section. Imaging-based prognostic features are summarized in Table 4.

**Table 4:** Main imaging-related factors associated with prognosis

Imaging-related factors	Specific data or values	Prognostic Significance
Tumor dimension	Longest diameter > 8 cm Volume > 200 ml	Reduced event-free survival
Tumor location	Axial skeleton (pelvis), intra-osseous location (versus extra-osseous)	Reduced overall-survival
(FDG-PET) SUVmax at baseline or after induction chemotherapy	+ 1 SUVmax unit = + 1 Hazard Ratio unit	Reduced overall-survival
Presence of distant metastasis at baseline imaging	Hazard Ratio range 2.4—4.4	Reduced overall-survival

## Surveillance—Role of MRI During Follow-up

The role of MRI in surveillance after initial treatment of Ewing sarcoma remains debated. In a cohort of 32 patients, Kasalak et al. reported local recurrence in 5 patients (15.6%), occurring between 2 and 7 months after completion of initial therapy [114]. Notably, all local recurrences were associated with concomitant metastatic relapse, and all affected patients died within 2 to 9 months following recurrence detection. These findings suggest that isolated local relapse detected by surveillance MRI is uncommon and that recurrence is frequently part of rapid systemic disease progression, raising questions regarding the impact of routine local MRI surveillance on overall survival.

## Treatment Response Assessment

Achieving a good histological response after neoadjuvant chemotherapy is a fundamental goal in Ewing sarcoma treatment. A good histological response is considered when ≥ 95% necrosis is found at the final histopathological analysis after excision. Good responders have significantly better clinical outcomes than bad responders [115]. Due to this, a pre-surgical imaging evaluation offering suggestions of histological response can be an important aid in clinical practice.

Conventional size-based criteria, such as RECIST (Response Evaluation Criteria in Solid Tumors) 1.1, are inadequate for evaluating response in Ewing sarcoma. Aghighi et al. studied 64 patients with newly diagnosed, non-metastatic Ewing sarcoma undergoing neoadjuvant chemotherapy and monitored by MRI (median follow-up 14.5 months, range 2–28) [116]. They found that relative reduction in longest diameter (1D (dimensional) RECIST) was not predictive of outcome in Cox regression analyses, whereas volume-based measurements, including 2D and 3D (ellipsoid or volumetric) assessments, were significantly associated with histological response and event-free survival (volume-based concordance-index [c-index] = 0.67, HR (hazard ratio) = 0.79, p = 0.001) [116].

Subsequent studies have sought to define imaging features predictive of histological response, defined as ≥ 95% tumor necrosis on surgical specimens. Aiba et al., in a cohort of 133 patients (median age 14 years, stages IIB–IIIB), developed a multivariate least absolute shrinkage and selection operator (LASSO) logistic regression model incorporating four imaging features: volumetric reduction, radiologic necrotic grade, complete regression of the extraskeletal component, and disappearance of peritumoral gadolinium enhancement. This model predicted histological response with high accuracy (AUC = 0.89) and was also significantly associated with overall survival (p = 0.029) [117].

Functional imaging with ^18^F-FDG PET/CT further contributes to response assessment. Annovazzi et al. analyzed 28 patients with non-metastatic osseous Ewing sarcoma, finding that a reduction in total lesion glycolysis (ΔTLG) of ≥ 60% predicted good histological response with 100% sensitivity and 77.8% specificity. Additionally, post-chemotherapy SUV > 3.3 or ΔTLG < −18% were independent predictors of poor histological response [118].

MRI with diffusion weighted imaging (DWI) has also been used to assess Ewing sarcoma neoadjuvant chemotherapy treatment response. In a study of 51 Ewing’s sarcoma patients, there was a significant change in the quantitative apparent diffusion coefficient (ADC) values before (0.71 ± 0.16) and after (1.6 ± 0.39) treatment were significant different (p < 0.001) [119]. Furthermore, this study demonstrated that ADC variations (ADC%) were significantly higher (p < 0.001) in the non-progressive group (128.3 ± 63.49%) compared to the progressive group (36.34 ± 78.7%), despite the fact that an increased ADC value was not always associated with a reduction in tumor volume.

Dynamic contrast-enhanced MRI (DCE-MRI) has also shown utility, with meta-analyses reporting a pooled sensitivity of 0.73 (95%CI: 0.54–0.88), a pooled specificity of 0.83 (95% CI: 0.67–0.94), and AUC = 0.839 for predicting histological response [120].

**Fig. 14:**
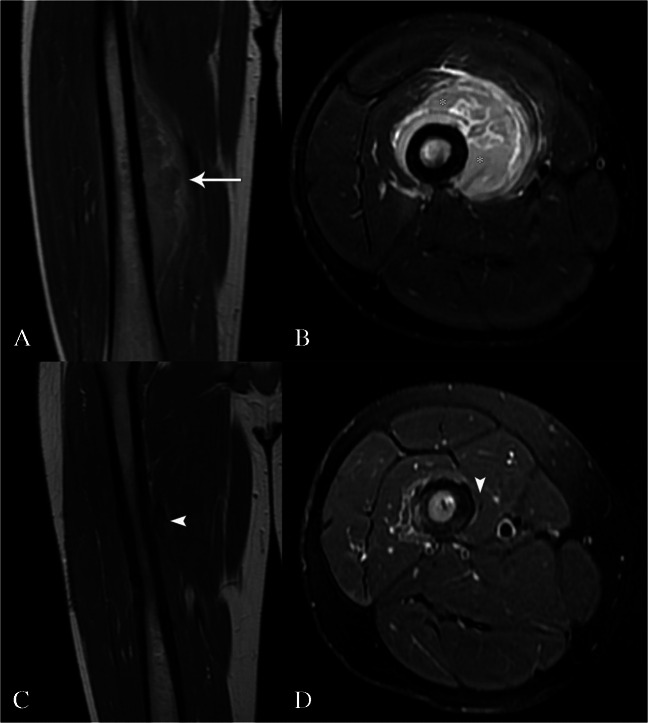
11-year-old male with Ewing sarcoma of the right femur diaphysis. Baseline coronal contrast enhanced T1 (**A**) and axial T2 FS (**B**) MRI of a femoral diaphyseal lesion with a large extraskeletal component (arrow and asterisks). MRI performed after neo-adjuvant chemotherapy, coronal contrast enhanced T1 (**C**) and axial T2 FS (**D**) reveal signs of good response with complete shrinkage of the soft-tissue mass (arrowheads) and volume reduction (> 65%). After surgical excision, the histopathological evaluation of necrosis on the whole tumor was excellent (100%)

Imaging features most strongly associated with histological response include volumetric reduction > 65% (Fig. 14) [121], disappearance of the extraskeletal tumor component, resolution of peritumoral enhancement, and reduction in metabolic activity on PET, as quantified by ΔTLG or SUVmax. These imaging biomarkers provide a non-invasive means to anticipate pathological response and may guide preoperative planning and risk-adapted therapeutic strategies (Table 5).

**Table 5:** Imaging features related to good histological response to chemotherapy (necrosis ≥ 95%)

Imaging-related changes after chemotherapy	Specific data or values
Tumor dimensions (volume)	Reduction > 65% of tumor volume from baseline
Tumor dimensions Contrast enhanced MRI Contrast enhanced MRI	Complete regression of the extraskeletal component of the tumor Disappearance of peritumoral enhancement areas Increase or appearance of necrotic areas inside the lesions
FDG PET	Reduction in total lesion glycolysis (ΔTLG) of ≥ 60%

Post-treatment surveillance of Ewing sarcoma remains a challenge. Relapse is associated with significant mortality, with 5-year survival in only 12–20% [122, 123]. Median survival time from detection is only approximately 12 months [122]. The vast majority of these, 70–80%, occur within the first two years [122]. It is unclear if early imaging truly improves survival [124]. Patients with asymptomatic, imaging-detected relapses enjoy a longer survival time (2.4 years compared to 1.2 years in relapses detected due to symptoms) [124]. This may, however, be secondary to lead-time bias from earlier detection and selection bias from less aggressive recurrence.

At present, consensus is to follow the primary site with MRI and evaluate for pulmonary metastases with chest CT, both at a frequency of every 3 months for two years, then every 6 months for the following three years [125–127]. Beyond that, imaging surveillance can be directed by patient symptoms or scheduled annual follow up. Whole-body imaging is often performed every 6–12 months, though we are unaware of studies comparing the optimal frequency of such imaging. While this was historically performed by bone scintigraphy, this modality is noted to have limited sensitivity to early disease [128]. PET/CT and whole-body MRI are more sensitive, but not without drawbacks [128–130]. PET/CT would significantly increase the total radiation dose of an entire surveillance regimen and whole-body MRI, at the time of this writing, is not widely accessible or reimbursed. Given similar sensitivity between these two modalities [129], we remain excited at the prospect of whole-body MRI having a role in early surveillance. More research, however, is needed to investigate whether this change would provide a survival benefit.

## Novel quantitative analyses (radiomics) and artificial intelligence (AI)

Radiomics enables the 3D extraction of quantitative metrics from segmented imaging data, capturing tumor features that are often imperceptible on conventional visual assessment [131]. These metrics can be used to develop predictive models for tumor classification (e.g., benign vs. malignant), grading, metastatic risk, local recurrence, treatment response, and overall prognosis [132].

In the context of Ewing sarcoma, radiomics has been applied to differentiate it from osteosarcoma in the pelvis and sacrum. CT-based radiomics achieved an AUC of 0.835, significantly outperforming radiologists’ performance (AUC = 0.757–0.811, p < 0.01), while MRI radiomics yielded an AUC of 0.881 on T2-weighted fat-saturated sequences and 0.765 on contrast-enhanced T1-weighted sequences [132–134]. Gitto et al. demonstrated that 3D MRI radiomics of skeletal Ewing sarcoma outperformed 2D approaches and predicted neoadjuvant chemotherapy response with 85% accuracy (AUC = 0.9) [135].

CT radiomics has also been used to predict pulmonary metastases in Ewing sarcoma, achieving an AUC of 0.843 [136], and to forecast postoperative recurrence, with training and validation cohort AUCs ranging from 0.820–0.922 and 0.759–0.880, respectively [137]. These findings underscore the potential of radiomics and artificial intelligence as non-invasive tools for personalized risk stratification and treatment planning in Ewing sarcoma.

## Conclusions

Ewing sarcoma is a highly aggressive small round cell sarcoma predominantly affecting children and adolescents, with a predilection for the diaphysis of long bones, pelvis, and, less commonly, flat bones or soft tissues. Accurate diagnosis relies on a combination of clinical, pathological, and molecular features, including the detection of characteristic FET-ETS fusion genes. Imaging plays a central role throughout the disease course, from initial diagnosis to treatment response assessment and surveillance. Conventional radiography and CT provide essential information on bone destruction patterns, periosteal reactions, and cortical involvement, while MRI offers superior delineation of intramedullary and soft tissue extension, skip metastases, and marrow involvement. Whole-body MRI and ^18^F-FDG PET/CT are increasingly employed for comprehensive staging, with PET/CT offering high sensitivity for osseous and nodal metastases and prognostic insights through quantitative SUV analysis with a higher availability.

Response to neoadjuvant chemotherapy is a critical prognostic factor, with histologic necrosis ≥ 95% strongly associated with improved survival. Imaging biomarkers, including tumor volume reduction, contrast enhancement patterns, and functional metrics from diffusion-weighted and dynamic contrast-enhanced MRI, as well as PET/CT-based metabolic response, provide valuable non-invasive indicators of histologic response. Furthermore, more recent quantitative approaches such as radiomics and artificial intelligence show promise in predicting tumor classification, chemotherapy response, metastases, and recurrence, potentially guiding individualized management strategies.

Overall, a multimodal imaging approach, integrated with clinical, histologic, and molecular data in a multidisciplinary environment of experts, is essential for optimal staging, treatment planning, and prognostic stratification in Ewing sarcoma.

## Data Availability

Not applicable.
